# Adsorption of Pb (II) Ions onto Hydroxyapatite Nanopowders in Aqueous Solutions

**DOI:** 10.3390/ma11112204

**Published:** 2018-11-07

**Authors:** Simona Liliana Iconaru, Mikael Motelica-Heino, Regis Guegan, Mircea Beuran, Adrian Costescu, Daniela Predoi

**Affiliations:** 1 National Institute of Materials Physics, Atomistilor Street, No. 405A, P.O. Box MG 07, 077125 Magurele, Romania; simonaiconaru@gmail.com; 2 ISTO, UMR 7327 CNRS Université d’Orléans, 1A rue de la Férollerie, 45071 Orléans CEDEX 2, France; mikael.motelica@univ-orleans.fr; 3 Faculty of Science and Engineering, Global Center for Science and Engineering, Waseda University, 3-4-1, Okubo, Shinjuku-ku, Tokyo 169-8555, Japan; regis.guegan@aoni.waseda.jp; 4 Carol Davila University of Medicine and Pharmacy, 8 Eroii Sanitari, Sector 5, 050474 Bucharest, Romania; beuranmircea@gmail.com; 5 Emergency Hospital Floreasca Bucharest, 8 Calea Floresca, 014461 Bucharest, Romania; 6 Faculty of Exact Sciences & Engineering, Hyperion University of Bucharest, 169 Calea Călărași, 030615 Bucharest, Romania; adrian.costescu@gmail.com

**Keywords:** hydroxyapatite nanoparticles, lead, water depollution

## Abstract

Contamination of water with heavy metals such as lead is a major worldwide problem because they affect the physiological functions of living organisms, cause cancer, and damage the immune system. Hydroxyapatite, (Ca_5_(PO_4_)_3_OH) is considered one of the most effective materials for removing heavy metals from contaminated water. The hydroxyapatite nanopowders (N-HAp) obtained by a co-precipitation method were used in this research to determine the effectiveness in removing lead ions from contaminated solutions. In this study, we have investigated the structure and morphology of N-HAp nanopowders using X-ray diffraction (XRD), electronic transmission microscopy (TEM), and scanning electron microscopy (SEM). The structure information was also obtained by spectroscopy measurements. The Fourier transform infrared spectroscopy (FTIR) and Raman spectroscopy measurements revealed the presence of peaks corresponding to the phosphate and hydroxyl groups. The ability of N-HAp nanopowders to adsorb lead ions from aqueous solutions were established. The results of the kinetic and equilibrium studies on the removal of Pb (II) from aqueous solution revealed that the adsorption of lead (II) cations is due to the surface reaction with the hydroxyl terminal groups on the adsorbent and the combination of the positive charges of the metal cations with the negative charges on the adsorbent surfaces. These observations could validate the use of these ceramic nanopowders in ecological remediation strategies.

## 1. Introduction

The economic development and rapid industrialization from recent years created many hazardous waste areas that contribute to the widespread contamination of soil and groundwater all over the world. These include often-abandoned mining sites, landfills, industrial areas, oil reservoirs, etc. The continued expansion of contaminated areas is a major environmental problem in the modern world. In this context, researchers around the world have turned their attention to finding efficient and cost-effective new methods of depollution, bearing in mind, that majority of pollutants such as heavy metals are not biodegradable and tend to accumulate in living organisms, inflicting serious damage to various vital functions and serious illnesses [1,2,3,4]. The conventional techniques studied so far and currently used in the environment rehabilitation have many disadvantages. Methods such as treating or aerating the soil, removing soil vapors or burning industrial residues used up until now have been reported to be expensive and to have a harmful impact on the ecosystem [5,6,7]. Because of these inconveniences, it is envisaged to develop technologies that will enable the environment to be rehabilitated using eco-friendly and cost-effective technologies. For the purpose of streamlining techniques for treating and preserving the environment, particular attention has been given to nanomaterials with biocompatible properties [8,9,10,11,12]. Numerous laboratory studies have been carried out in recent years on various materials with potential applications for the conservation and treatment of the environment. Among the studied materials, special attention was granted to hydroxyapatite-based composites for the purpose of decontamination of soils, ocean and/or continental waters. Even though they are well known for applications in the biomedical field, apatites possess a strong affinity for heavy metal sorption [10,11,12,13,14,15,16]. Currently, many studies have been focused on the development of apatites with mesoporous structure and high specific surface area, which gives them a high capacity to adsorb heavy metals from contaminated waters and soils. Existing literature studies involving the adsorption properties of porous and poorly crystalline apatites are very rare. Interaction of apatites with both metals and radionuclides is an ongoing research topic. The adsorption mechanisms presented by various laboratory investigations vary depending on the adsorbed cation. Suzuki and co-workers, [17] in their studies on the mechanism of adsorption of ions such as Pb^2+^, Cu^2+^, Cd^2+^, Zn^2+^, Ni^2+^, Co^2+^, Mn^2+^, Mg^2+^, and Ba^2+^ explained their adsorption by apatite as the result of an ion exchange. During the years, numerous models have been developed to obtain a detailed description of the metallic ions adsorption onto different kind of materials [18,19,20]. F. Di Natale et al. [19] in their studies describe a model for metallic ions adsorption from aqueous solutions using activated carbons, while Erto et al. [20] in their paper regarding the adsorption of cadmium and zinc onto activated carbon, emphasize that the most adequate model to predict adsorption data in the case of cadmium and zinc adsorption on activated carbon is by using the Extended Langmuir model. Moreover, their results showed that in the case of cadmium adsorption better results were obtained using the Vacancy Solution Theory (VST).

Middelburg and Comans [21] in their studies on the use of hydroxyapatite for Cd^2+^ sorption described the heavy metal transport model in hydroxyapatite through sorption reactions and transport physical processes. The immobilization of lead from aqueous solutions with hydroxyapatite has been also described by Xu and Schwartz [22] as being achievable as a result of the dissolution/precipitation mechanisms occurring during the adsorption process. The data obtained by now has concluded that the use of nanoparticle-based nanostructured materials [23,24] can lead to the development of far more efficient technologies for decontamination of soils and waters at much lower costs compared to techniques based on micro- and/or macroscopic materials. Due to its high porosity and affinity to heavy metal ions, apatite and calcium phosphate materials are considered some of the most promising materials to obtain effective environmental technologies.

The aim of this study was to obtain hydroxyapatite nanoparticles with a high affinity for heavy metal ions. The physico-chemical properties of hydroxyapatite nanopowders N-HAp, obtained by coprecipitation have been investigated using XRD, TEM, SEM, FTIR, and Raman. The affinity towards lead ions of the N-HAp nanopowders has been studied by batch adsorption experiments.

## 2. Materials and Methods

### 2.1. Synthesis of Hydroxyapatite Nanoparticles (HAp)

The hydroxyapatite, nanoparticles, Ca_10_(PO_4_)_6_(OH)_2_, were obtained using an adapted coprecipitation method which was previously detailed in [25,26].

The reagents for synthesis, calcium nitrate [Ca(NO_3_)_2_·4H_2_O] and ammonium dihydrogen phosphate [(NH_4_)_2_HPO_4_] were purchased from Alpha Aesare, Karlsruhe, Germany. The Ca_10_(PO_4_)_6_(OH)_2_, with x = 0 (HAp), nano powder was synthesized by an adapted co-precipitation method (Ca/P molar ratio: 1.67) using [(NH_4_)_2_HPO_4_] and Ca(NO_3_)_2_·4H_2_O [27]. The hydroxyapatite nanoparticles was effectuated by adjustment the atomic ratio Ca/P as 1.67. A designed amount of Ca(NO_3_)_2_·4H_2_O were dissolved in deionised water to obtain 400 mL Ca-containing solution. In the same time, a designed amount of ammonium dihydrogen phosphate (NH_4_)_2_HPO_4_ was dissolved in deionised water to make 400 mL P-containing solution. The two solutions were agitated at 100 °C during 40 min. The P-containing solution was added drop by drop into the Ca-containing solution. The pH of resulting mixture was kept constant during the reaction by adding NH_3_ and stirred continuously for 4 h. The resulting final mixture was filtered and washed several times with deionized water. Finally, the recovered powder was dried in an oven at 100 °C for 72 h. The resulting powder after drying was used in the experiments in this study.

### 2.2. Structural and Morphological Characterizations

The structure and morphology of the hydroxyapatite nanoparticles obtained by coprecipitation were characterized by XRD, SEM, EDX and TEM. Investigations of XRD were performed for the N-HAp nanopowders with a Bruker D8 Advance diffractometer (Bruker, Karlsruhe, Germany) having a nickel filtered Cu K*α* (λ = 1.5418 Å) radiation and a high efficiency one-dimensional detector (Lynx Eye type, Bruker, Karlsruhe, Germany) operated in integration mode. The measurements were recorded over the 2θ range of 20–80°, using a step size of 0.02° and 34 s measuring time per step. The morphology and the elemental composition of the N-HAp nanopowders was investigated by scanning electron microscopy (SEM) using a HITACHI S4500 microscope (Tokyo, Japan) equipped with an X-ray Energy Dispersive Spectroscopy (EDX) system.

Furthermore, the size and morphology of the N-HAp nanopowders were analyzed using transmission electron microscopy (TEM) with a CM 20 (Philips—FEI, Eindhoven, Netherlands), equipped with a filament Lab6 that works at 200 kV. For TEM observation, the N-HAp nanopowders were dispersed in ethanol using an ultrasonic bath (Retsch GmbH, Haan, Germany) and a drop of the resulting solution was deposited on a carbon-coated Cu grid and left to dry for 24 h at room temperature before visualization. Information about the specific surface area, pore volume, pore size and particle size of the samples was obtained by adsorption-desorption (nitrogen, N_2_) gas analysis using Brunauer–Emmett–Teller (BET) method using an ASAP 2020 (Micromeritics Instrument Corp, Norcross, GA, USA) instrument.

### 2.3. Batch Adsorption Experiments

The efficiency of lead adsorption onto hydroxyapatite nanopowders was investigated by batch adsorption experiments. The batch adsorption experiments were performed using silicon tubes with aqueous solutions containing lead ions in a concentration range of 0–100 mg L^−1^. The contaminated lead solution were obtained using Pb(NO_3_)_2_ (Alpha Aesar, Alfa Aesar, Karlsruhe, Germany; 99% purity) and distilled water. The amount of the N-HAp nanopowders used, as adsorbent was 0.2 g and the solution pH of the initial contaminated solution was adjusted to a value of 5 ± 0.4 using a 0.1 M hydrochloric acid (HCl) solution. During the batch adsorption experiments, the solution volume was kept at 20 mL and the mixture was stirred on a Mixer SRT1 Roller (Stuart Scientific, Staffordshire, UK) for 48 h. After 48 h, the tubes were centrifuged for 1 h at 10,000 rpm. The supernatant was filtered, recovered and analyzed by Flame Atomic Absorption Spectrometry (AAS) using a Zeeman HITACHI Z-8100 from Japan Hitachi (Tokyo, Japan). The AAS measurements were conducted under a constant air flow rate at a wavelength of 283.3 nm according to the operational condition for lead. The batch experiments were carried out at room temperature, in triplicate.

## 3. Results and Discussions

**Figure 1:**
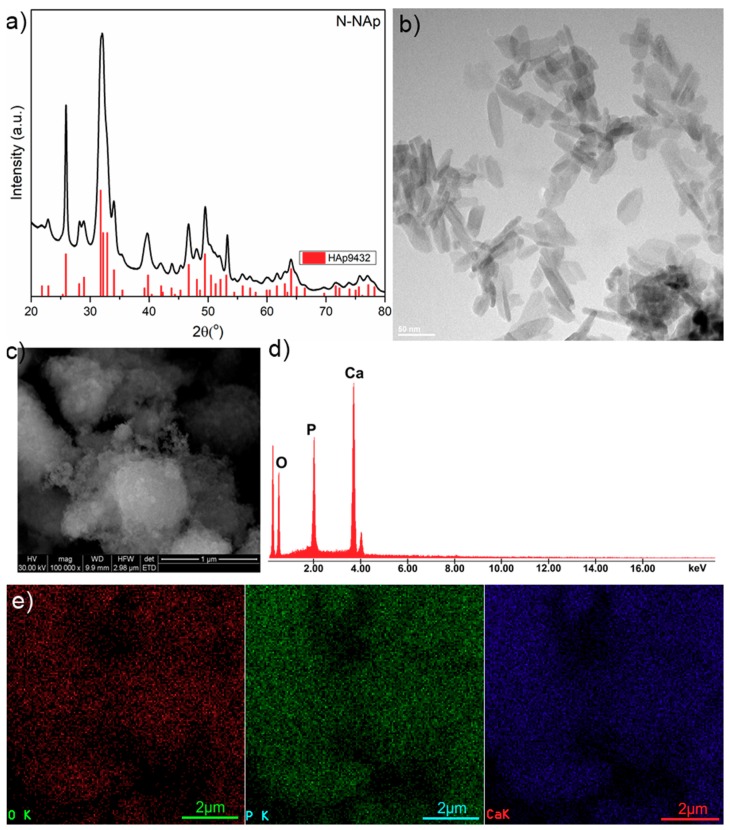
XRD patterns of hydroxyapatite nanopowders (N-HAp) nanopowders and ICDD-PDF (International Center for Diffraction Data- Powder Diffraction File) 9-432 (**a**); TEM image of N-HAp nanopowders (**b**); SEM micrograph of N-HAp (**c**); EDX spectra of N-HAp nanopowders (**d**); elemental mapping of N-HAp nanopowders (**e**).

The structure, morphology and elemental analysis were presented in Figure 1. In order to highlight the crystalline structure of the obtained hydroxyapatite samples, XRD measurements were performed. Furthermore, in order to obtain conclusive results, N-HAp nanopowders were also analyzed by TEM. For the morphological characterization and determination of the elemental composition of the N-HAp samples, an electronic scanning microscope equipped with an EDX system was used. Figure 1a shows the diffraction patterns of N-HAp nanoparticles obtained by co-precipitation. The TEM image for the N-HAp powders are presented in Figure 1b. SEM micrographs and EDX spectra for N-HAp powders are also shown in Figure 1c,d, respectively.

As it can be seen from Figure 1a, the peaks occurring in the diffraction patterns of N-HAp are characteristic to hexagonal hydroxyapatite in agreement with the ICDD-PDF (International Center for Diffraction Data- Powder Diffraction File) 9-432, indicating that the analyzed powders exhibit a characteristic HAp hexagonal structure [28,29,30,31,32]. Moreover, the diffraction patterns characteristic to the theoretical diffraction patterns of the hexagonal hydroxyapatite (ICDD-PDF 9-432) are also shown in Figure 1a (in red). Furthermore, no additional peaks were found in the diffraction pattern of N-HAp. The TEM image (Figure 1b) revealed that the hydroxyapatite particles obtained by co-precipitation are of nanometric dimensions and ellipsoidal shape. The SEM analysis of the N-HAp particles revealed that they have nanometric size and ellipsoidal shape in accordance with the information obtained from TEM images (Figure 1c). From the EDX spectrum (Figure 1d), it can be observed that only the constitutive elements of hydroxyapatite (calcium (Ca), phosphorus (P), and oxygen (O)) have been highlighted in the analyzed sample. The elemental mapping of N-HAp nanopowders (Figure 1e) demonstrated that the constituent elements were evenly distributed in the samples.

**Figure 2:**
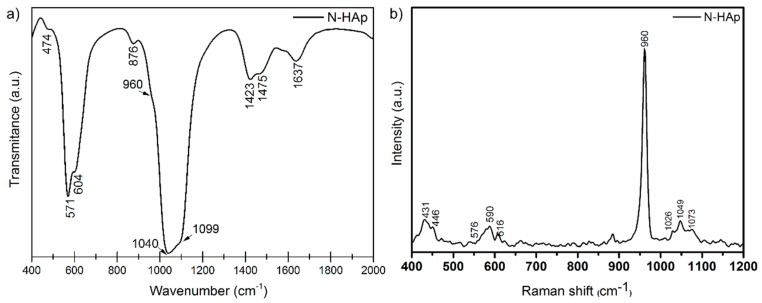
FTIR (**a**) and Raman (**b**) spectra of N-HAp nanopowders.

FTIR was used for the determination of the functional groups of N-HAp nanopowders [27,33,34,35]. The analyzed N-HAp FTIR spectrum was performed in the range of 400–2000 cm^−1^ with a resolution of 4 cm^−1^ and 256 scans (Figure 2a). In order to obtain complementary information, the N-HAp powders were also investigated using Raman spectroscopy (Figure 2b). The Raman spectrum was carried out in the range of 400–1200 cm^−1^. Figure 2a presents the FTIR spectra of N-HAp nanopowders obtained in transmission mode. The spectrum reveals the presence of the vibrational modes corresponding to phosphate and hydroxyl groups. The main vibrational bands characteristic to phosphate groups were observed at 474 cm^−1^, 571 cm^−1^, and 604 cm^−1^. The clear presence of these three bands confirms the formation of hydroxyapatite. The vibration band corresponding to the HPO_4_^2−^ group was also observed at 876 cm^−1^. The vibrational bands corresponding to the surface adsorbed water were highlighted in the 1600–1700 cm^−1^ interval. The Raman spectrum recorded for the N-HAp powders are shown in Figure 2b. It can be observed that the vibration bands characteristic to PO_4_^3−^ are present in the N-HAp sample spectrum in accordance with previous studies [34,35]. In the Raman spectrum, the band at 960 cm^−1^, associated with the internal vibration modes of ν_1_ (PO_4_^3−^), (symmetrical stretching of the links P–O) can be clearly observed. The vibrational bands observed at 1026 cm^−1^, 1049 cm^−1^ and 1073 cm^−1^ could be attributed to the elongation of the asymmetric link PO (ν_3_) while the vibrational bands from 576 cm ^−1^ or 590 cm^−1^ and 616 cm^−1^ are due to the vibration modes (ν_4_) of the phosphate group. The bands observed at 434 cm^−1^ (ν_2_) and 431 cm^−1^ (ν_2_) are assigned to O–P–O [34,35] bonds.

In order to obtain information regarding the specific surface area, pore volume, pore size, and particle size, gas adsorption/desorption studies have been carried out. The specific surface area (71.97 m^2^/g) of the N-HAp powders was calculated using the BET (Brunauer, Emmet, Teller) method [36]. Moreover, the Barrett–Joyner–Halenda (BJH) analysis results for N-Hap samples were shown in Table 1.

The BET method [36] used to determine the specific surface of the samples is based on a proposed Brunauer, Emmett, and Teller theory and assumes that the surface is homogeneous and the adsorption is carried out in several layers, the molecules of the first layer serve as an adsorption site for the second layer. Moreover, during this process it is assumed that there is a permanent balance between the number of molecules adsorbed to the surface and those that are desorbed. Often, the BET equation is applicable only to a certain relative pressure range (between 0.05 and 0.1), region in which the theoretical and experimental curves coincide, as the BET theory does not take into account the heterogeneity of the solid material. On the other hand, the Barret–Joyner–Halenda (BJH) [37] model used is based on the condensation of nitrogen in mesopores that occurs at a pressure lower than the saturation vapor pressure of the absorbent. More of that, the BJH model uses the classical Kelvin law derived from the Laplace equation. Furthermore, it is well known that there is no unique definition of “pore diameter” or “pore size”. Each method of determining the pore size defines a pore size in terms of a pore model that is most appropriate for the quantity measured in the experiment. In this study, flame atomic absorption spectroscopy measurements were used to measure lead ion concentration in order to assess the efficiency of the N-HAp powders in the removal of lead ions from aqueous solutions.

**Figure 3:**
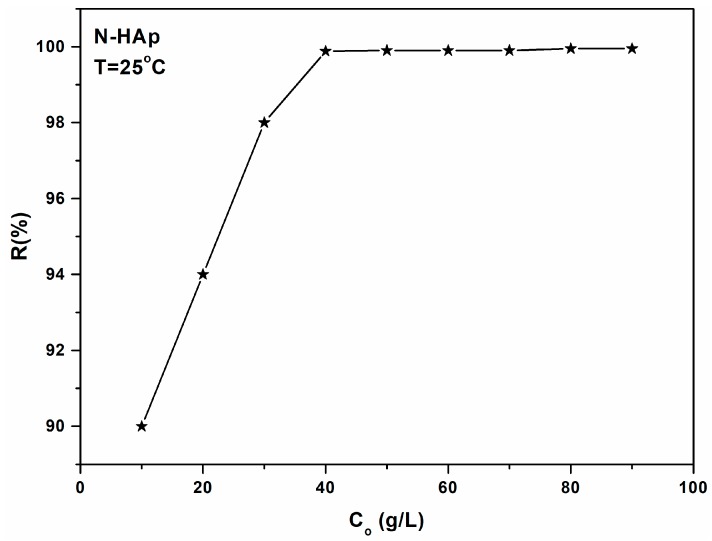
The effect of the initial concentration of Pb^2+^ on the N-HAp efficiency in the removal of lead ions from aqueous solutions.

For that purpose, the hydroxyapatite nanoparticles (N-HAp) were mixed with solutions having different concentrations of Pb^2+^ (0–100 mg·L^−1^) and a pH value of 5. Figure 3 shows the efficiency of N-HAp nanopowders in the removal of lead ions from aqueous solutions. It can be seen that the lead removal efficiency depends on the initial concentration of Pb^2+^. For a lead concentration of 20 mg L^−1^, the removal efficiency of lead ions from the contaminated solution of N-HAp powders was 94%, thus demonstrating that the removal efficiency are dependent on the amount of the adsorbent, and on the initial Pb^2+^ concentration.

For lead concentrations in the range of 40–100 mg∙L^−1^, it could be seen that the removal efficiency of Pb^2+^ ions was about 99.2%. The removal efficiency of lead ions onto N-HAp nanopowders was calculated using the following formula:

(1) $$R\left(\%\right)=\frac{\left(C_{o}-C_{e}\right)}{C_{0}}\times 100\;$$

where C_o_ and C_e_ are the initial and equilibrium concentrations of Pb^2+^ (g/L) ions.

**Figure 4:**
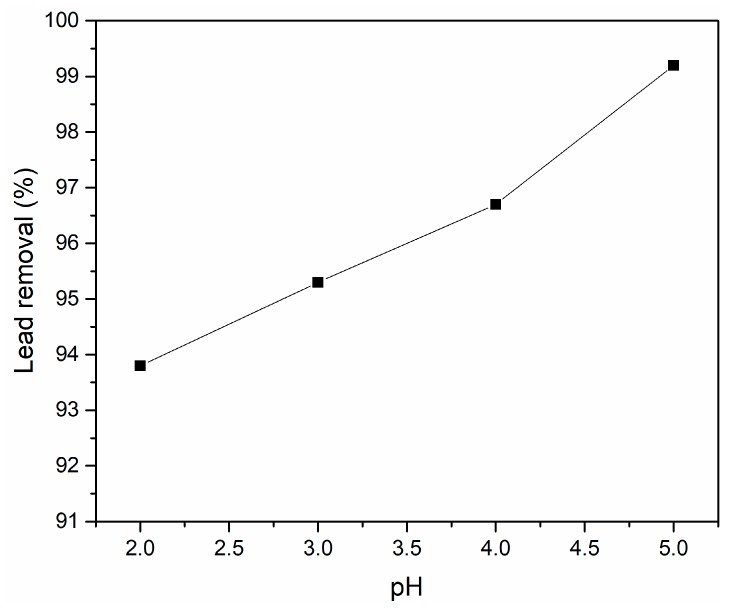
Effect of the solution pH on the removal of Pb^2+^ ions by N-HAp powders.

For a complex understanding of the adsorption process, the effect of the pH of the initial lead solution was considered and batch experiments at four different pH values were undertaken. The results of the batch adsorption experiments for an initial concentration of Pb^2+^ of 50 (g/L) are presented in Figure 4. This investigation has revealed that the pH value of the initial contaminated solution had an influence on the lead adsorption process onto N-HAp. According to the results, a removal efficiency of 99.2% was achieved for pH 5. On the other hand, the removal efficiency of lead ions by N-HAp powders was 96.7% at pH 4. For pH 3 and 2 the achieved removal efficiency was 95.3% and 93.8%. These results have emphasized that even though the efficiency of Pb^2+^ ions from aqueous solution was influenced by the pH of the initial solution, at all the tested pH values, the removal efficiency was greater than 93%. Similar results regarding the influence of the pH value of the initial solution on the adsorption of lead from aqueous solutions were obtained by Cozmuta et al. [38]. Cozmuta et al. reported in their study that the decrease of pH induced a decrease of the maximum adsorption capacity of lead ions onto Na-clinoptilolite. The effect of the pH on the lead adsorption was also reported by Zhang et al. [39] in their studies regarding the “effect of temperature, salinity, and pH on the adsorption of lead by sediment of a tidal river in east China”. The results presented by Zhang et al. emphasized an increase of Pb adsorption on sediment with the increase of pH values from 1 to 4. These results confirm that lead adsorption is strongly influenced by the initial pH value of the contaminated solution.

In order to better understand and describe the processes of adsorption of metal ions on different materials, over the years, a wide range of models have been developed and employed: Langmuir; Freundlich; Brunauer–Emmett–Teller; Redlich–Peterson; Dubinin–Radushkevich; Temkin; Toth; Koble–Corrigan; Sips; Khan; Hill; Flory–Huggins; and Radke–Prausnitz [40]. The kinetic aspect plays an important role in all the models listed above. Thus, the adsorption equilibrium is defined as a dynamic equilibrium state, with both adsorption and desorption rates [41]. The physico-chemical parameters taken together with basic thermodynamic assumptions can provide an understanding of the adsorption mechanisms, surface properties, and adsorbent affinity [42]. To describe the adsorption process of lead ions on N-HAp powders, the Langmuir and Freundlich adsorption models were used [43].

In this study, the adsorption isotherms were obtained by mixing solutions containing different concentrations of Pb^2+^ with a known amount of N-HAp powders until thermodynamic equilibrium at ambient temperature (T = 25 °C) was achieved. The adsorption capacity, defined as the amount of metal retained per mass unit, was estimated. Also, the quantity of Pb^2+^ ions adsorbed at equilibrium per unit mass, Q_e_, was determined using the formula:

(2) $$\;Q_{e}=\frac{\left(C_{0}-C_{e}\right)}{m}\;$$

where C_o_ (mg/L) is the initial metal ion concentration, C_e_ (mg/L) is the equilibrium concentration of Pb (II), V (L) is the volume of the solution, and m (g) is the mass of the adsorbent.

The Langmuir adsorption isotherm was originally developed to describe the gas-solid adsorption phase in activated carbon, and is most often used to quantify and emphasize the efficiency of a variety of biosorbents [41]. The empirical model involves the monolayer adsorption (the adsorbed layer has the thickness of a molecule). Adsorption can take place at a finite number of localized and defined areas that are identical and equivalent [44,45]. From a graphical point of view, the Langmuir isotherm is characterized by a saturation zone [46]. Thus, the theoretical Langmuir isotherm is often used to describe the adsorption of a dissolved solution from a liquid solution as follows [47,48]:

(3) $$\;Q_{e}=\frac{q_{m}K_{L}C_{e}}{1+K_{L}C_{e}}\;$$

where q_m_ and K_L_ are the Langmuir constants, representing the maximum adsorption capacity, and the constant energy associated with the heat of adsorption. The two Langmuir constants can be determined using the graphical representation (C_e_/Q_e_) function of (C_e_) from the linear form of the Langmuir Equation:

(4) $$\frac{C_{e}}{Q_{e}}=\frac{1}{\left(q_{m}\times K_{L}\right)}+\frac{C_{e}}{q_{m}}\;$$

**Figure 5:**
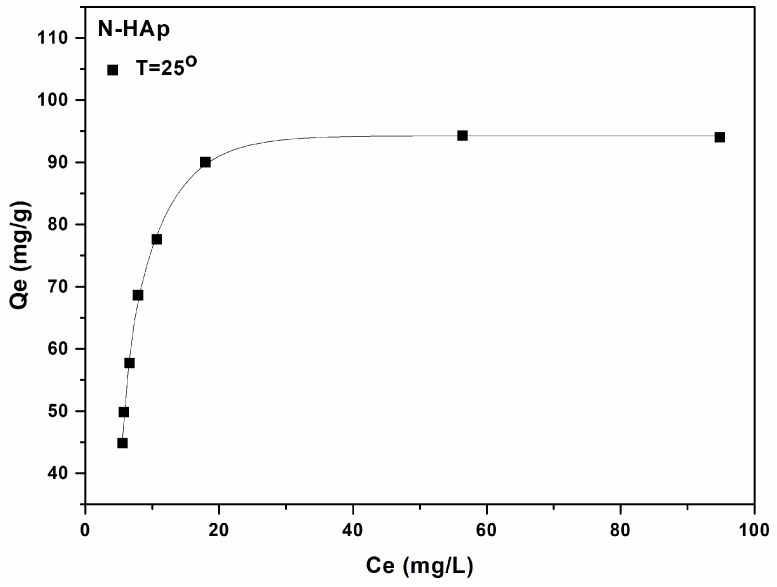
Graphic representation of the amount of material adsorbed at equilibrium by the equilibrium concentration for the adsorption of Pb^2+^ ions on N-HAp powders.

Figure 5 shows the experimental data as well as the theoretical Langmuir model when the N-HAp nanopowder was used for the adsorption of Pb^2+^ ions from aqueous solutions. The graphical representations of the Pb^2+^ adsorbed ions on the mass unit by N-HAp powders, (q_e_) depending on the concentration of Pb^2+^ remaining in solution (C_e_) are shown in Figure 5.

Figure 5 shows the graphical representation of C_e_/Q_e_ function of C_e_. The data has revealed that at ambient temperature, the R^2^ coefficient deduced from the Langmuir isotherm is equal to 0.997 for N-HAp. In good agreement with previous studies [49,50], the transformation of the nonlinear isothermal equation into the linear form by a nonlinear method has not raised any problem. The two Langmuir constants were determined from the graphical representation of (C_e_/Q_e_) function of (C_e_). Numerous studies have been reported in the literature on the ability of apatites and hydroxyapatite-based materials to adsorb metal ions from aqueous solutions. Depending on the experimental conditions of the adsorption process like pH solution, sorbent mass, initial concentration of pollutant material and the experiment duration, the adsorption capacity of Pb^2+^ ions by hydroxyapatite was reported between 84 and 620 mg·g^−1^ [51]. Following the reaction of N-HAp with solutions contaminated with Pb^2+^ ions, the lead ions were removed from the solutions and the hydroxyapatite dissolved. The Langmuir, q_m_ and K_L_ constants, representing the maximum adsorption capacity, and the constant energy associated with the heat of adsorption, were also determined using the graphical representation of the linear form of the Langmuir equation.

The results obtained from the adsorption batch experiments to remove lead ions from aqueous solutions using N-HAp powders have shown that N-HAp powders have been very effective in removing lead ions from the contaminated aqueous solutions. Thus, for the N-HAp samples an adsorption capacity of 99.31 mg (Pb)/g, and a K_L_ coefficient value of 2.35 L/mg, was obtained. For a better understanding of the mechanisms involved in the removal of lead ions by N-HAp powders, the Freundlich model was also used. Freundlich adsorption isotherm [52] is the first known relationship that describes a non-ideal and reversible adsorption mechanism, which is not limited to monolayer formation. This empirical model can be applied in multilayer adsorption with uneven distribution of heat of adsorption and affinity of heterogeneous surfaces [53]. Historically, it was developed for the adsorption of animal-derived coal, demonstrating that the mass adsorbent ratio offered by the adsorbent and solutions is not constant at a different concentration of the solution [54]. From this perspective, the adsorbed amount is the sum of the adsorption in all areas, with the stronger bonding zones being occupied first, and the adsorption energy decreases exponentially at the time of the adsorption process [55].

The Freundlich isotherm is described by the following Equation:

(5)  Qe=kf×Ce1n 

where Q_e_ is the amount of material adsorbed at equilibrium (mg/g), C_e_ is the metal ion concentration at equilibrium (mg/L) and k_f_ [mg/g (mg/L)^−1/n^] and n are Freundlich constants, representing the adsorption capacity and the adsorption intensity of the adsorbent.

The two Freundlich constants can be determined from the graphical representation (lnQ_e_) function of (lnC_e_) from the linear form of the Freundlich Equation:

(6) $$\;{\mathrm{lnQ}}_{e}={\mathrm{lnk}}_{f}+\frac{1}{n}{\mathrm{lnC}}_{e}\;$$

**Figure 6:**
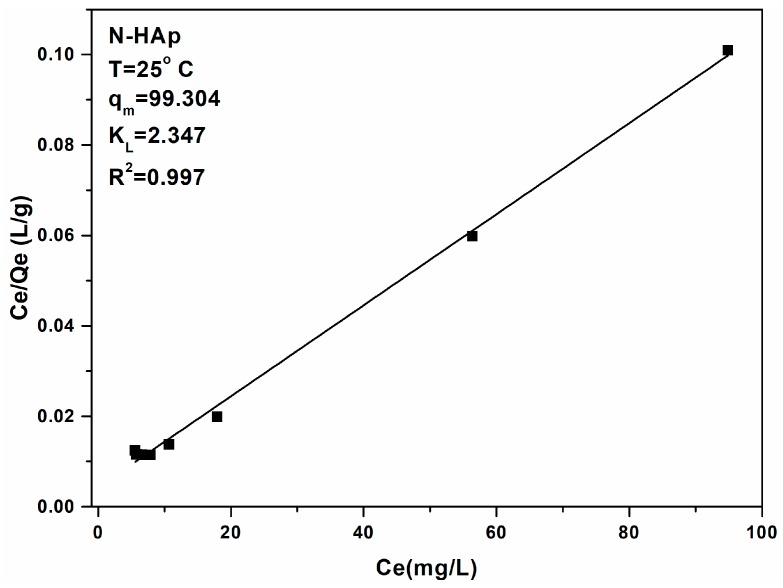
Langmuir linearized fits for the adsorption of lead ions on N-HAp.

**Figure 7:**
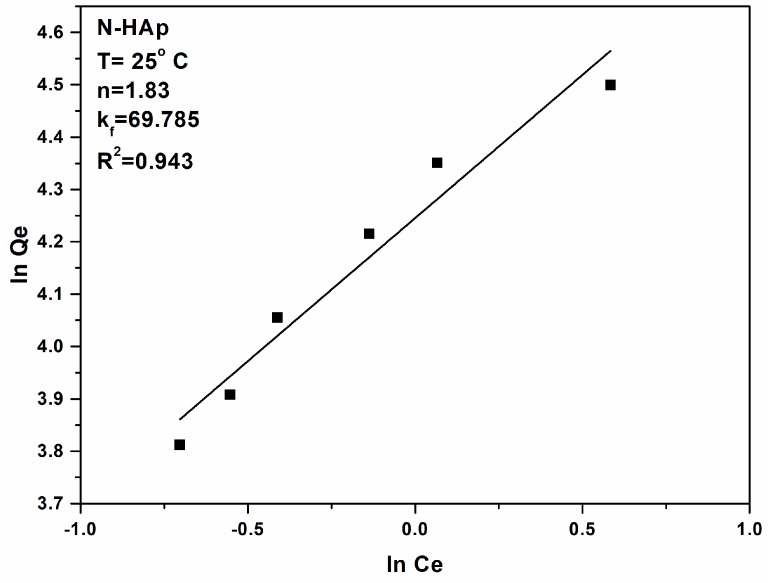
Freundlich linearized fits for the adsorption of lead ions on N-Hap.

Figure 6 shows the graphical representations of (lnQ_e_) function of (lnC_e_) for the lead ion adsorption experiments on N-HAp powders. The k_f_, Freundlich constant, is an indicator of the adsorption capacity of the materials used as adsorbent, while 1/n is a function of the power adsorption from the process [56]. Figure 7 presents the Freundlich linearized fits for the adsorption of lead ions on N-HAp powders.

If n = 1, then the separation of the two phases is independent of the concentration. If 1/n is below 1 this indicates a normal adsorption process. On the other hand, if 1/n is less than 1, this indicates a cooperative adsorption process [57]. According to the literature if the value of n is between one and ten, this indicates a favorable adsorption process [58]. The values obtained for n from the linearized form of the Freundlich equation (Figure 7), for the lead ion adsorption experiments on N-HAp powders were greater than 1 leading to a value of 1/n < 1 thus signifying a normal adsorption process. Table 2 shows the values for both Langmuir and Freundlich constants obtained in the experiments to remove Pb^2+^ ions from aqueous solutions using N-HAp powders.

**Figure 8:**
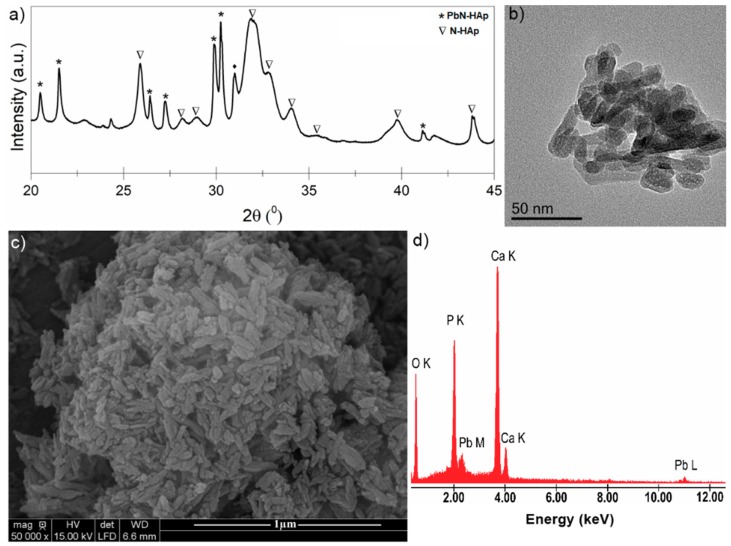
XRD patterns of N-HAp after lead immobilization (**a**); TEM image N-HAp after lead sorption (**b**); SEM image N-HAp after lead sorption (**c**); EDX spectra of N-HAp nanopowders (**d**).

According with previous studies [59], the separation factor R_L_ indicates the shape of isotherm, when R_L_ > 1, the isotherm is unfavorable, when R_L_ = 1, it is linear; when between 0 and 1, it is favorable and when R_L_ = 0 it is irreversible. In this study, the R_L_ values were between 0 and 1 indicated a favorable adsorption of Pb^2+^ on the adsorbent. It has also been observed that the uptake of Pb (II) throughout the process was high, due to the affinity that is relatively large between Pb (II) and N-HAp. Comparing the correlation coefficients (R^2^), it can be deduced that the experimental equilibrium data of Pb (II) sorption was best suited to the Langmuir model. This behavior may be due to the homogeneous reaction on the surface for Pb (II) sorption. It was clearly seen that N-HAp was favorable for adsorption of Pb (II). The data obtained from adsorption isotherms of Pb (II) agreed with the Langmuir model. Furthermore, XRD analysis confirmed that the main mechanism in the adsorption of Pb (II) was due to the partial dissolution of N-HAp and pyromorphite reprecipitation. Similar results have been reported by Xu et al. [60], in their studies regarding lead immobilization by hydroxyapatite in aqueous solutions. The studies conducted by Xu et al. have revealed that the lead removal process using hydroxyapatite is kinetically fast. Moreover, they have emphasized that for a lead concentration of 100 mg·L^−1^ Pb^2+^ the dominant reaction mechanisms involved in the adsorption process were the dissolution of hydroxyapatite and the precipitation of lead apatites such as hydroxypyromorphite in systems with no chloride, and of chloropyromorphite in systems that contain chloride [60]. The X-ray diffraction (XRD) patterns shown in Figure 8a evidenced the structural changes of N-HAp after Pb (II) adsorption. A mixed phase of pyromorphite (Pb_10_(PO_4_)_6_(OH)_2_) and HAp was revealed. In the TEM image was observed that the particles after sorption of lead have a more rounded shape (Figure 8b). More, from TEM image, it was observed that after the sorption of lead the particles increased their size and were crowded. The SEM image (Figure 8c) also showed agglomerated ellipsoidal particles in agreement with TEM image. The presence of lead in the recovered powder after the sorption of lead from the contaminated solution was also evidenced in the EDX spectrum (Figure 8d).

Kinetic and equilibrium studies on the removal of Pb (II) from aqueous solution [61] have shown that the adsorption of lead cations (II) is due to the surface reaction with the hydroxyl terminal groups on the adsorbent and the combination of the positive charges of the metal cations with the negative charges on the adsorbent surfaces. The adsorption of Pb (II) ions by HAp can take place through various mechanisms. Functional groups, surface properties, structure are just a few factors that can affect the preference of adsorption. Moreover, the sorbate properties such as ionic size, molecular structure, ionic charge or concentration can also influence adsorption. On the other hand, the chemistry of tha solution such as the pH and ionic strength plays an important role in the adsorption mechanism. According to previous studies [19,20,62] it can be assumed that the adsorption properties of HAp nanopowders was mainly due to the functional groups Ca^2+^, PO_4_^3−^, and OH^−^ involved in the surface reactions. The previous studies [22,63,64] suggested that the first mechanism is the adsorption of Pb (II) ions on the HAp surfaces [22] that this mechanism is followed by ion exchange reaction between Pb (II) ions adsorbed and Ca^2+^ ions of HAp [22].

Previous studies [22,65,66,67,68] confirmed that the main mechanism responsible for Pb (II) adsorption by HAp was the dissolution of HAp followed by precipitation of pyromphoritis (a more stable phase than HAp). According to these precedent studies [22,65,66,67,68], reactions could be described as follows:

The dissolution of HAp has been described as such:

Ca_10_ (PO_4_)_6_(OH)_2_ + 14H^+^ → 10Ca^2+^ + 6HPO_4_^−^ + 2H_2_O (7)

The pyromphoritis precipitation could be described as follows:

10Pb^2+^ + 6H_2_PO_4_^−^ + 2H_2_O → 4H^+^ +Pb_10_(PO_4_)_6_(OH)_2_ (8)

In agreement with the studies previously presented by Q.Y. Ma et al. [69] regarding the in situ lead immobilization by apatite, during dissolution/precipitation process there may be a proton exchange between the adsorbent and the metal ions in the solutions. So, the ≡POH site of HAp can transform into a ≡POPb^+^ site.

Taking into account previous studies [70,71], we could say that adsorption of Pb (II) ions on the HAp surface which leads to replacement of Ca(II) could be attributed to the adsorption of Pb (II) by HAp and can be described as follows:

Ca_10_(PO_4_)_6_(OH)_2_ + *x*Pb^2+^ → Ca_10−x_Pb_x_(PO_4_)_6_ + *x*Pb^2+^ (9)

Z. Zhang et al. [72], in their studies on immobilization of lead and cadmium from aqueous solution and contaminated sediment using nano-hydroxyapatite, have shown that electronegativity and ionic radii in the range of 0.90–1.30 Å could be two other factors that influence the sorption capacity of HAp (ionic radius of Ca^2+^ is 0.99 Å). The high sorption capacity obtained for lead could be explained by the high electronegativity of Pb^2+^ (2.33) and the ionic radiation value of 1.20 Å. The results of this study were consistent with the the results presented by A. Corami et al. [73] in their studies on cadmium removal from single- and multi-metal (Cd + Pb + Zn + Cu) solutions by sorption on hydroxyapatite.

Our study is part of the overall effort to obtain effective adsorbents in the removal of heavy metal ions [74,75,76]. Hydroxyapatite being a non-toxic material has attracted attention due to its low cost and excellent adsorption properties for environmental applications. However, much effort has to be done to obtain materials that will increase the efficiency of removing heavy metal ions from aqueous systems as they pose a serious threat to public health and the ecological system due to high toxicity.

## 4. Conclusions

A nano hydroxyapatite was successfully synthesized and used for Pb (II) removal from aqueous solutions. The physico-chemical characterization of hydroxyapatite nanopowders obtained by an adapted coprecipitation method was explored. The obtained products presented a structure characteristic to the hexagonal hydroxyapatite and no additional peaks were found in the diffraction spectra of N-HAp. The morphology of N-HAp was nanometric size with ellipsoidal shaped particles. The efficiency of Pb (II) removal was analyzed by adsorption experiments. The removal efficiency of Pb^2+^ ions was approximately 99.2% for lead concentrations in the range of 40–100 mg. The coefficient of regression (R^2^) that was obtained using the Langmuir model was higher compared to that obtained using the Freundlich model. The results presented in this study could be useful for developing materials for environmental applications. Moreover, the material used as an adsorbent for removing heavy metals from contaminated waters is an ecological material that can be obtained at low costs.

## Figures and Tables

**Table 1:** Barrett-Joyner-Halenda (BJH) analysis results for N-Hap samples.

N-HAp Information from BJH Analysis		N-HAp
Cumulative pore surface area (Adsorption) obtained by the BJH method (m^2^/g)		91.3
Cumulative pore surface area (Desorption) obtained by the BJH method (m^2^/g)		100.1
Pore volume	Total pore volume (Absorption) (cm³/g)	0.025
	Total pore volume (Desorption) (cm³/g)	0.4
Pore size	Average pore size (Absorption) (nm)	13.6
	Average pore size (Desorption) (nm)	22.6
	The average pore diameter (Absorption) obtained by the BJH method (nm)	18.23
	The average pore diameter (Desorption) obtained by the BJH method (nm)	16.66
Particle size (nm)		83.37

**Table 2:** Langmuir and Freundlich isotherm parameters for Pb^2+^ adsorption onto N-HAp nanopowders.

Sample	Langmuir			Freundlich		
N-HAp	R^2^	q_m_ (mg/g)	K_L_ (L/mg)	R^2^	n	k_f_
	0.997	99.304 ± 0.2	2.347 ± 0.2	0.943	1.83 ± 0.3	69.785 ± 0.3
